# Hydroxytyrosol Exerts Anti-Inflammatory and Anti-Oxidant Activities in a Mouse Model of Systemic Inflammation

**DOI:** 10.3390/molecules23123212

**Published:** 2018-12-05

**Authors:** Raffaela Fuccelli, Roberto Fabiani, Patrizia Rosignoli

**Affiliations:** Department of Chemistry, Biology and Biotechnologies, University of Perugia, del Giochetto Street, 06123 Perugia, Italy; raffaela.fuccelli@unipg.it (R.F.); roberto.fabiani@unipg.it (R.F.)

**Keywords:** hydroxytyrosol, inflammation, mice, oxidative stress, prevention

## Abstract

Hydroxytyrosol (3,4-dihydroxyphenil-ethanol, HT), the major phenol derived from olive oil consumption, has shown different anti-inflammatory and anti-oxidant activities in vitro which may explain the chronic-degenerative diseases preventive properties of olive oil. The aim of this study was to examine the ability of HT reduce inflammatory markers, Cyclooxygenase-2 (COX2) and Tumour Necrosis Factor alfa (TNF-α and oxidative stress in vivo on a mouse model of systemic inflammation. Balb/c mice were pre-treated with HT (40 and 80 mg/Kg b.w.) and then stimulated by intraperitoneal injection of lipopolysaccharide (LPS). Blood was collected to measure COX2 gene expression by qPCR and TNF-α level by ELISA kit in plasma. In addition, the total anti-oxidant power of plasma and the DNA damage were measured by FRAP test and COMET assay, respectively. LPS increased the COX2 expression, the TNF-α production and the DNA damage. HT administration prevented all LPS-induced effects and improved the anti-oxidant power of plasma. HT demonstrated in vivo anti-inflammatory and anti-oxidant abilities. The results may explain the health effects of olive oil in Mediterranean diet. HT represents an interesting molecule for the development of new nutraceuticals and functional food useful in chronic diseases prevention.

## 1. Introduction

Chronic inflammation and oxidative stress are involved in the pathogenesis of many chronic-degenerative diseases such as diabetes and cancer, in addition to cardiovascular, autoimmune and neurodegenerative diseases [1]. 

Chronic inflammation is characterised by an overproduction of pro-inflammatory molecules, such as Tumour Necrosis Factor alpha (TNF-α) and Cyclooxygenase-2 (COX2) which persist during chronic diseases [2]. TNF-α is a key pro-inflammatory cytokine which initiates the inflammatory processes and modulates the immunity system; therefore, its unregulated production or its expression in an inappropriate site can lead to pathogenic consequences [3]. Indeed, persistently elevated levels of TNF-α have been showed in chronic inflammation and associated with a variety of autoimmune diseases. The block of the TNF-α signalling has been a target to treat various inflammatory diseases and successfully used in the case of autoimmune diseases such as rheumatoid arthritis, Crohn’s disease, and psoriasis [4]. COX2 is an inducible enzyme stimulated by growth factors, oncogenes, tumour promoters or inflammatory cytokines [5]. COX2 plays a key role in the release of prostaglandin E2 (PGE2) that regulates multiple biological processes under physiological and pathological conditions including neuronal functions, female reproduction, vascular hypertension, kidney function, gastric mucosal protection, pain hypersensitivity, inflammation, tumour grown and metastasization [6].

The overproduction of reactive oxygen species (ROS) during oxidative stress induces damage to the important cellular macromolecules including DNA [7]. Single- and double- strand breaks (SSD and DSB respectively) are often incremented in most chronic-degenerative diseases [8]. In addition, the overproduction of ROS can also induce an inflammatory response which, in turn, contributes to a further increase the DNA damage [8].

Despite significant advances in therapies for the treatment of many chronic-degenerative diseases, novel approaches are still required for their prevention. The interest on dietary supplements and nutraceuticals as support of the classical pharmacotherapy is growing. In addition, plant-derived compounds represent potential molecules that may be used for the development of new drugs designed for the treatment and/or control of chronic inflammatory states. Current epidemiological and experimental studies support a beneficial role of dietary polyphenols in several chronic diseases [9,10]. Polyphenols derived from the olive tree, leaves and oil, have shown potent in vitro antioxidant and anti-inflammatory properties. In particular, hydroxytyrosol (3,4-dihydroxyphenyl-ethanol, HT), which is present in olive oil both as free compound and linked to the dialdehydic form of elenoic acid (3,4-DHPEA-EDA and pHPEA-EDA) has been deeply investigated since it is the main phenolic end-product of their metabolism. It showed evident anti-inflammatory, anti-thrombotic, and anti-atherogenic properties [11,12,13]. It is also able to contrast hyperglycemias, insulin resistance and the metabolic syndrome, improving endothelial function, haemostatic and lipid profiles [14] and decreasing oxidative stress [15,16]. In addition, HT showed further positive health effects such as anti-steatotic and pro-autophagic properties and improves mitochondrial function [17]. We recently reported that HT was able to inhibit the production of O_2_^−^, the expression of COX2 and the release of PGE2 in human freshly isolated peripheral blood monocytes while it increased the TNF-α production [18]. The study of molecular mechanisms involved in these effects showed that the low level of PGE2 after LPS stimulation in monocytes exposed to HT was responsible of the low level of cAMP required to decrease TNF-α secretion [19]. Similarly, non-steroidal anti-inflammatory drugs (NSAIDs), such as celecoxib, inhibit the COX2 activity and increase the TNF-α production in PBMC stimulated with LPS and this effect was reversed by exogenous addition of PGE2 [20]. These evidences suggest that HT could act as COX2 inhibitor. On the base of these considerations, the objectives of the present study were to evaluate the in vivo potential preventive effects of HT on LPS-induced increment of: (a) COX2 gene expression; (b) TNF-α secretion; (c) oxidative DNA damage. In addition, the effect of HT on the plasma antioxidant power of mice treated with LPS has been also investigated. 

## 2. Results

### 2.1. HT Inhibits the COX2 Gene Expression in LPS-Stimulated Mouse Model

**Figure 1:**
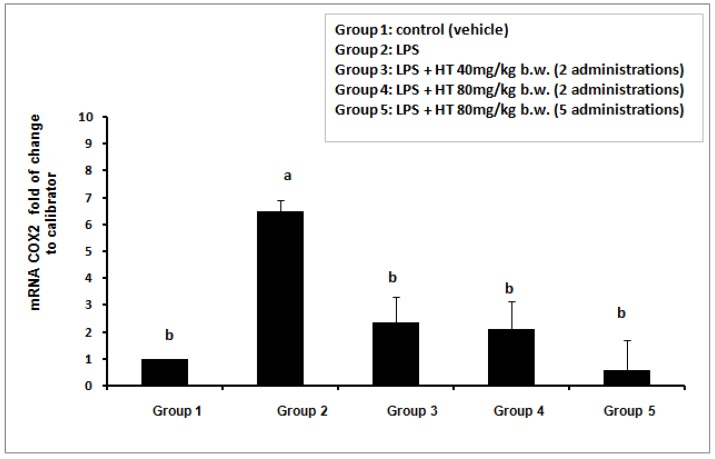
Effect of HT on LPS-induced COX2 gene expression (mRNA) evaluated by qPCR in mice. Total RNA was extracted from 100 µL of blood opportunely stored. Data represent the increment of COX2 mRNA in respect to calibrator (vehicle group). Each bar represents the mean ± S.D. of values obtained from the 8 mice/group. Bars with a different letter are significantly different, *p* < 0.05.

When mice did not receive any stimulus (control group = vehicle) low level of COX2 gene expression in whole blood cells PBMC was evidenced (Figure 1). The exposure of animals to LPS (50 µg/mouse) (group 2) caused a strong activation of COX2 gene expression (increment is about 6 times with respect to the control). The pre-treatment with HT by oral gavage was able to significantly suppress the up-regulation of this pro-inflammatory gene at all doses tested. The mRNA value is reduced to levels not statistically different from the control group even at the lowest dose used. Increasing in HT dose to 80 mg/kg b.w. and prolonging the HT treatment (80 mg/kg b.w. for 5 administrations) further reduced the mRNA COX2 level although in a statistically not significant manner (Figure 1).

### 2.2. HT Reduces the TNF-α Cytokine Secretion in LPS Stimulated Mouse Model

**Figure 2:**
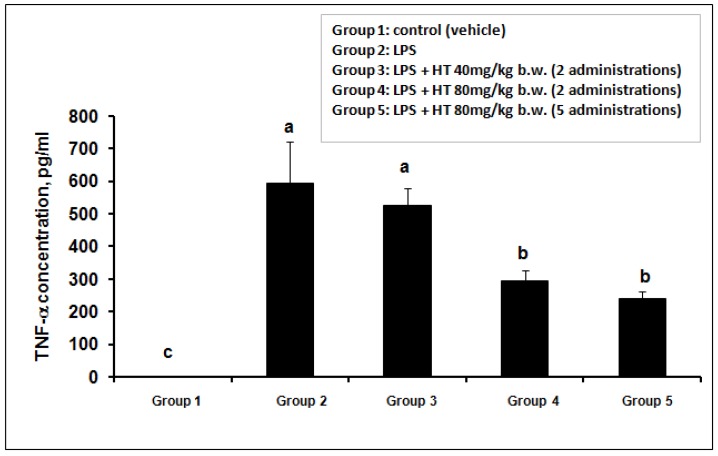
Effect of HT on LPS-induced TNF-α production in mice plasma. 50 µL/mouse of plasma were used to determine the TNF-α concentration by ELISA kit according to the manufacturer’s instruction. Values are represented as pg/ml. Each bar represents the mean ± S.D. of values obtained from the 8 mice/group. Bars with a different letter are significantly different, *p* < 0.05.

The basal level of TNF-α in mice of the control group (vehicle) was very low (0.011 +/− 0.0061 pg/mL, data not shown). The LPS treatment (50 µg/mouse) resulted in a prompt elevation of this pro-inflammatory cytokine. In fact, two hours after LPS injection, plasma concentration of TNF-α in LPS-exposed mice reached a value of 597 ± 124 pg/mL. The pre-treatment of animals with HT at the lower dose (HT 40 mg/kg b.w.) did not reduce this value in a statistically significant manner. High doses of HT (80 mg/kg b.w. and 80 mg/kg b.w. for 5 administrations) were able to decrease the LPS-induced TNF-α production by about 50% (Figure 2). 

### 2.3. HT Improves the Antioxidant Power of Plasma in LPS Stimulated Mouse Model

**Figure 3:**
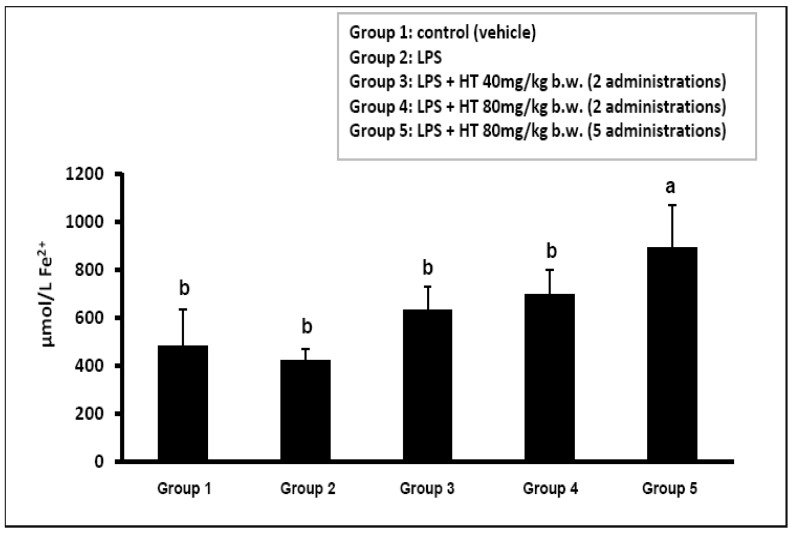
Effect of LPS and HT on antioxidant power of plasma in mice. 100 µL/mouse of plasma were used to determine the antioxidant ability of plasma by FRAP test. Data are expressed as µmol/liter of Fe^2+^ Each bar represents the mean ± S.D. of values obtained from the 8 mice/group. Bars with a different letter are significantly different, *p* < 0.05.

The plasma antioxidant power in the different mice groups were determined by the FRAP assay. The results showed in figure 3 indicate that plasma antioxidant power was not influenced by the LPS treatment while it was increased by HT even if the statistical significant effect was reached only at the highest dose for the prolonged treatment time (80 mg/kg b.w. for 5 administrations). In this last case the antioxidant power of plasma doubled the basal value (*p* < 0.01) (Figure 3).

### 2.4. HT Prevents the DNA Damage Induced by LPS-Stimulation in Mouse Model

**Figure 4:**
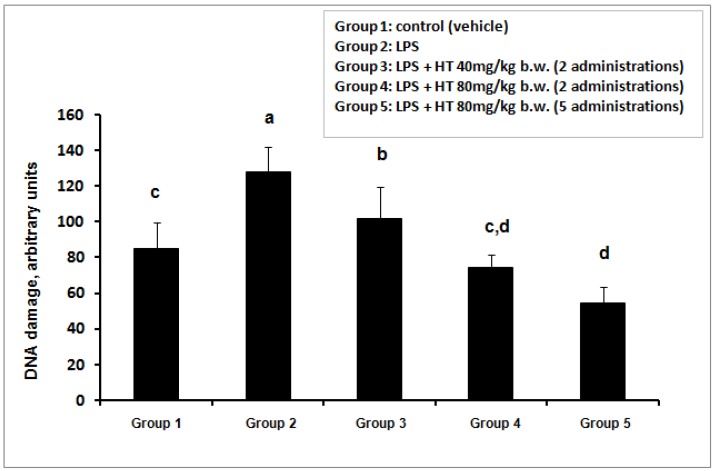
Effect of HT on DNA damage in whole blood cells.10 µL of cell suspension were included in 75 µL of low melting point agar and used for the comet assay. Data are expressed as arbitrary units as described in Materials and Methods Section. Each bar represents the mean ± S.D. of values obtained from the 8 mice/group. Bars with a different letter are significantly different, *p* < 0.05.

The genotoxic effects of LPS i.p. injection and the effect of HT on blood cells are shown in Figure 4. The DNA damage was quantified immediately after the sacrifice of mice. The whole blood cells of mice in the control group (vehicle) showed a moderate level of DNA damage which was significantly increased by the exposure to LPS (85 A.U. vs 128 A.U., respectively). The HT pre-treatment prevented this damage in a dose dependent manner. It should be underlined that the highest dose of HT completely prevented the DNA damage evoked by LPS and further reduced the DNA damage under the basal level (63 A.U. with HT 80 mg/kg b.w. vs 85 A.U. with vehicle).

## 3. Discussion

Chronic-degenerative diseases are the leading global cause of death and are responsible for 70% of deaths worldwide representing an important public health challenge in all countries [21]. Recent researches have shown that both inflammation and oxidative stress are involved in their pathogenesis. Over the past few years, there has been a growing interest in “nutraceuticals”, which have been investigated for application toward different types of non-communicable diseases [22]. This approach takes advantage of anti-inflammatory and anti-oxidant properties of naturally occurring agents which originate from vegetables, spices and fruits. The traditional Mediterranean diet represents an important source of molecules of plant origin, such as HT, as HT, a major phenolic metabolite resulting from the consumption of extra virgin olive oil (EVOO). The antioxidant and anti-inflammatory properties of HT has been widely investigated in vitro and less in in vivo animal models [23]. In this paper we have studied the antioxidant and anti-inflammatory properties of HT in a mouse model of systemic inflammation induced by LPS i.p. injection. Our results are of particular importance since this procedure mimes’ pathophysiologic conditions similar to those observed in human inflammatory process [24]. In agreement with our results on the LPS induction of COX2 gene expression, previous investigations have demonstrated that mouse treatment with i.p. injection of LPS caused an increase of COX2 level in liver cells [25]. In addition, COX2 increment was also observed both in lung cells after intra-tracheally administrated LPS [26] and in diaphragm tissue in LPS induced peritonitis [27]. In this study we also observed that the in vivo pre-treatment with HT 40–80 mg/kg b.w. reduces the COX2 expression. The HT doses selected in this work take into account the results obtained in studies on similar animal models in which the absence of adverse effects and efficacy in various biological markers has been reported [28,29,30,31]. It should be emphasized that, although these doses may seem high compared to the food intake, they can be used for the formulation of food supplements and fortified foods. The HT inhibition of COX2 gene expression was also demonstrated in other experimental models. For instance, in a rat model of rheumatoid arthritis HT-supplemented refined olive oil administered by gavage significantly down-regulated the COX2 expression in knee joint specimens [32]. Sanchez-Fidalgo et al. observed a reduction of COX2 gene expression in a DSS-induced acute colitis in mice treated with extra-virgin olive oil enriched with HT [33,34] and HT-acetate [35]. In this contest, it should be underlined that non-steroidal anti-inflammatory drugs (NSAIDs), which act via inhibition of COX2, are among the most widely used medications worldwide for treatment of both acute or chronic pain and inflammation. However, their use is usually associated with a large range of side effects especially against cardiovascular and gastrointestinal systems [36]. Thus, strategies to mitigate the risks and maximize the therapeutic benefits associated with NSAIDs should continue to be employed. One of such strategies may be use lowest effective dose of drugs in combination with natural products. In this respect, an approach has been made to treat rheumatoid arthritis with a dietary flavanol Morin (2′,4′,3,5,7-pentahydroxyflavonoid) present in guava leaves, onion, apples, and Moraceae group. Arthritic rats treated with combination of Morin and indomethacin exhibited lower level of COX2 mRNA than arthritic rats treated only with indomethacin [37]. In addition, the strategy of combining molecules to increase their effects has also been tested against tumour diseases; in this regard recently it was demonstrated, both in vitro and in vivo in animal models, that a combination of different polyphenols improves their anti-tumoral properties [38,39]. Based on our results, it could be assumed that HT could be used in innovative formulation of new supplements and/or in the production of fortified foods with anti-inflammatory activity. Further studies will be necessary to investigate its possible long-term side effects although Auñon-Calles et al. demonstrated that HT showed no adverse effects up to 500 mg/kg/d in rats treated for 13 weeks [40].

The ability of LPS i.p. injection to increase the TNF-α level in serum of mice was reported [41]. Large amounts of TNF-α are responsible for the perpetuation of inflammation [42]. Moreover, the increased production of TNF-α by NSAIDs, exacerbating the pro-inflammatory environment, could be responsible for the side effects associated with their prolonged use [20]. Therefore, compounds that inhibit TNF-α production are considered beneficial for the treatment of many inflammatory diseases. In the present study, we demonstrated for the first time that pre-treatment with HT was able to decrease the TNF-α level in plasma of mice exposed to LPS i.p. injection. 

The modulation of TNF production by HT, both as single molecule and in mixture with other olive oil phenols or added in a standard diet, was studied both in vitro and in vivo in different experimental models with contrasting results. In human monocytes THP-1 cell lines, the pre-treatment with HT did not reduce the LPS-induced TNF production [24]. In the same cells, on the contrary, Zhang et al. demonstrated that HT was able to decrease the level of TNF-α inhibiting its gene expression [43]. Similar results were observed in murine macrophages RAW264.7 [12]. Previously, in human PBMC exposed to LPS in combination with different doses of HT we showed an increment of the TNF-α production [18]. Data from in vivo studies concerning the effect of HT on TNF are also contrasting. When HT was added to diet failed to reduce the TNF-α production in paw homogenate in a murine collagen-induced arthritis [44] and in colon tissue homogenate in a chronic DSS colitis model [33]. An inhibition of TNF-α production was observed in plasma of mice exposed to a hypercolesterolemic diet enriched with HT [45] as well as in serum of high-fat diet fed mice receiving HT 5mg/day for 12 weeks [46]. An HT reduction of TNF-α at protein and gene expression level has been also showed by Wu and coll. in a mouse model of mastitis obtained by Staphylococcus aureus infusion into the mammary glands [47]. Moreover, the authors underlined some anti-inflammatory properties of HT including the reduction of both mieloperoxidase enzyme (MPO) and cytokines IL-1 and IL-6 into the mammary tissue [47]. On the other hand, when HT was administered by i.p. injection as polyphenols mixture (Phenolea Active Complex) it was observed an increment of TNF-α level in a mouse model of paw inflammation [48]. Probably, these differences could be related to the different administration protocols: i.p. injection vs oral gavage. Further experiments are needed to explain these contrasting results. 

Regarding the anti-inflammatory effects exerted by HT directly in human, it should be underlined that most investigators used an extra virgin olive oil (EVOO) enriched with different polyphenols, including HT. These intervention studies mainly showed beneficial effects of enriched olive oil on several key processes related to cardiovascular risk [49,50]. In other studies, it was shown the ability of a multicomponent nutraceutical containing HT to reduce free radicals and oxidized cholesterol, and to increase the serum antioxidant capacity [51]. An olive leaf extract (OLE) containing HT was used in a randomised, controlled, double-blind, crossover intervention trial to test its anti-inflammatory abilities in pre-hypertensive male volunteers. The data demonstrated a reduction in interleukin-8 [52]. To date, only one study has been carried out to investigate the anti-inflammatory effects of HT, as single compound, on human [53]. In this last study, human healthy volunteers were treated for 3 weeks with gastro-resistant capsules of HT (Fenolia™, P&P Farma Srl, Turin, Italy) and several biomarkers of inflammation and oxidative stress such as thiol group, total antioxidant status, superoxide dismutase-1 resulted increased by HT treatment while nitrite, nitrate, and malondialdehyde (MDA) were drastically reduced [53]. A previous work performed with Hytolive® (Genosa, Madrid, Spain), an olive mill wastewater (OMWW) extract selectively enriched in HT, did not demonstrate an effect on TNF-α production [54]. Due to the lack of adequate data, further human intervention studies with HT will be of great importance. 

Regarding the effects exerted by LPS in mice, we also observed an evident increment of DNA damage in blood cells. This finding is supported by previous studies showing that in vitro treatment of human PBMC with LPS resulted in a marked oxidative DNA damage [55]. Accordingly, Serebrovska et al. have found that LPS increased reactive oxygen species (ROS) in rat’s blood as measured by lucigenin- and luminol-enhanced chemiluminescence [56]. As suggested by the authors, LPS may initiate a positive feedback loop, involving cytokine expression, neutrophil and macrophage attraction, NAD(P)-H oxidase activation and ROS production [56]. On this base, it is possible that HT could inhibit one or more of these pathways, as suggested by its ability to decreased, in vitro, the production of O_2_^−^ and PGE2 in monocytes [18]. In contrast with our findings, a previous study in which DNA strand breaks were evaluated by the comet assay in mouse leukocytes after LPS stimulation, a small but not statistically significant increment of DNA damage was observed in the animals treated with LPS [57]. However, it should be underlined that the experimental conditions were different with respect to both the LPS dose (2.5 mg/kg b.w. used by us vs 5 mg/kg b.w.) and time of treatment (2 h used by us vs 6 h and 15 min) [57].

Interestingly, we also observed for the first time a complete prevention of LPS-induced DNA damage in animals treated with HT along with an improvement of the antioxidant potential of plasma. These results support our previous finding showing the ability of this compound, both by itself and when present in complex mixtures, to prevent the DNA damage induced by pro-oxidant molecules in PBMC treated in vitro [58,59,60]. 

Our results showed that LPS treatment did not reduce the antioxidant potential of plasma. Other authors also did not observe any reduction on FRAP value 1 h or 5 h after LPS treatment [61]. The lack of the reduction of antioxidant potential of plasma could be due to the short time spent between the LPS i.p. injection and the recovery of plasma. Indeed Kim et al., although in a different experimental model, observed a decrement of FRAP value after 12 h from the LPS treatment [62]. It should be also considered that FRAP test is not able to measure all the radicals produced during oxidative stress. Moreover, we found that the treatment with HT at highest dose increased the FRAP level respect to the control group. A similar improvement of the plasma antioxidant potential was also reported in a rat model by other phenols isolated from cranberries [62] and acanthus ilicipholius [63]. In contrast, no effects on FRAP were observed in rats treated with resveratrol [61]. 

## 4. Materials and Methods

### 4.1. Animals

Nulliparous nonpregnant BALB/c mice of 9-week-old (Harlan Laboratories, S. Pietro al Natisone, Udine, Italy) were maintained in Plexiglas cages under standardized conditions. The animal body weight range was 19–21 g. All efforts were made to minimize and reduce animal suffering and for limiting the number of animals used. The animal experiments were carried out following the procedure described by the guidelines of the European Community Council Directive of 24 November 1986 (86/609/EEC). The animal care and experimental protocols were performed according to the Italian Approved Animal Welfare Assurance. The approval protocol number was DR 33/2011-B, released by “Ministrero del Lavoro, della Salute e delle Politiche Sociali” (Rome, Italy) in 3 March 2011. Mice consumed diet deprived of polyphenols (Mucedola, S. Pietro al Natisone, Milan) and water ad libitum for 7 days to allow the acclimatisation. 

### 4.2. Experimental Design

**Scheme 1:**
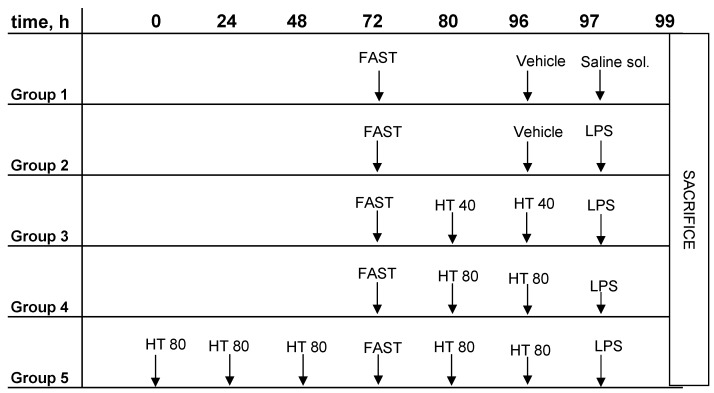
Diagram of the protocol design for the study of HT anti-inflammatory and anti-oxidant activities in the mouse model of LPS-induced systemic inflammation. BALB/c mice were divided randomly in 5 groups (8 animals/group). After acclimatisation, animals fed diet without polyphenols for 3 days during which only the group 5 received daily HT 80 mg/kg b.w. by oral gavage. After that, all animals were kept fast (time 72 in the scheme) for the remaining period of the trial during which they received only the gavage with HT twice (time 80 and 96 in the scheme). After 1 h from the last gavage animals received an i.p. injection containing either saline solution (group 1) or LPS 50 µg/mouse (groups 2, 3, 4 and 5). All mice were sacrificed 2 h after i.p. injection and blood were collected by intracardiac puncture for the subsequent assays.

The experimental procedure was synthesized in Scheme 1. To test the effects of HT (Cayman Chemical Company, Ann Harbor, MI, USA, 320 mM in ethanol stock solution) the following experiment was performed: 40 animals were divided in 5 experimental groups: group 1 (control, n = 8), group 2 (LPS, n = 8), group 3 (HT 40 mg/kg b.w., n = 8), group 4 (HT 80 mg/kg b.w., n = 8), group 5 (HT 80 mg/kg b.w. administrated for 5 times, n = 8). The sample size was determined fixing a level of significance of 5% and a power of study of 90% and considering as variance and expected difference results previously obtained by in vitro studies. After acclimatisation, animals fed diet without polyphenols for 3 days during which only the group 5 received daily HT 80 mg/kg b.w. by oral gavage. After these 3 days, all animals were kept fast (time 72 h) for the whole period of the trial during which they were treated with HT by gavage. Two administrations were carried out (after 8 h and 24 h from start of fasting) with vehicle (ethanol 6%) in animals of group 1 and 2, with HT (40 mg/kg b.w.) group 3 and HT (80 mg/kg b.w.) groups 4 and 5. 1 h after the last gavage all animals received intraperitoneal (i.p.) injection with LPS (50 µg/mouse) except the group 1 that received saline solution. Finally, 2 h after i.p. all animals were sacrificed. 

### 4.3. Animals Sacrifice

Two hours later the LPS treatment mice were anesthetized with Pentothal injection (200 µL/mouse, 6 mg/mL) and blood was collected by intra-cardiac puncture with heparinized syringes. The blood obtained was divided into two aliquots: 100 µL were sampled in RNA protect Animal Blood Tubes containing a solution that immediately stabilizes cellular RNA (QiagenS.r.l. Milan, Italy) and stored at −20 °C until the analysis of COX2 gene expression. The remaining aliquot was centrifuged at 4000 rpm × 15 min. The resulting plasma was stored at 4 °C and used to determine the TNF-α cytokine level and the total antioxidant power, while the cell pellets were resuspended in 500 µL of RPMI medium and immediately used for the assessment of DNA damage.

### 4.4. COX2 mRNA Analysis by Real-Time PCR

Total RNA was extracted from blood cells using a dedicated kit (Qiagen, RNeasy Protect Animal Blood Kit) according to the manufacturer’s instructions. RNA concentrations were measured spectrophotometrically at 260 nm, and the purity was determined by the A260/A280 ratio. Samples were stored at −80 °C before use. For cDNA synthesis, 1 μg of total RNA was reverse-transcribed using a high capacity cDNA reverse transcription kit (Qiagen, Quantitec Reverse Trascription kit) according to the manufacturer’s instructions. cDNA was stored at −20 °C prior to real-time PCR. Gene expression was quantified by real-time quantitative PCR (qRT-PCR) using a Stratagene Mx3000P Q-PCR machine (Agilent Technologies, La Jolla, CA, USA) starting from aliquots of 2 µL of cDNA. Reactions were performed in triplicate using Quantitec SYBR Green PCR Kit (QiagenS.r.l. Milan, Italy) and Quantitect primer assay specific for COX2 and β-actin, used as internal control, according to the manufacturer’s instructions. For each PCR run, a negative control was systematically added, in which RNase-free water replaced cDNA. In addition, a single peak was observed for each of the products by melting curve analysis, performed routinely in all samples after amplification. No product was detected in the absence of RT in any assay for any of the products, thus indicating that there was no genomic contamination. The relative quantities of target gene mRNA were measured by following a ΔΔCt method employing an amplification plot (fluorescence signal vs. cycle number). ΔCtwas calculated subtracting the average Ct value of β-actin to the average Ct value of COX2 gene for each sample. ΔΔCt is the difference between the ΔCt for each sample and the ΔCt of vehicle-treated cells as control. The results were reported as fold of change to calibrator (group 1 = control) sets equal to 1.

### 4.5. TNF-α Cytokine Determination

TNF-α was analysed in the plasma of mice using a commercially available ELISA kit (Quantikine ELISA mouse TNF-α Immunoassay by R&D Systems, Minneapolis, MN, USA) according to the manufacturer’s instruction. TNF-α was determined from a standard curve. The concentrations were expressed as picograms per milliliter and all assays were performed in duplicate which were averaged for statistical comparisons.

### 4.6. DNA Damage Assessment 

The DNA damage was determined in blood cells of mice immediately after sacrifice by the single cell gel electrophoresis assay (SCGE or comet assay) as previously described [64]. 10 µL of cell pellet resuspended in RPMI were transferred to 1.5 mL Eppendorf tubes, mixed with 75 µL of low melting agarose [0.7% in phosphate buffer (PBS)] and distributed onto conventional microscopic slides precoated with normal melting agarose (0.5% in PBS) and dried at 50 °C. After the agarose was solidified (4 °C for 5 min), a second layer of low melting agarose was applied similarly to the previous. The slides were then immersed in the lysis solution (2.5 M NaCl, 100 mM Na_2_EDTA, 10 mM TrisHCl pH 10.0, containing freshly added 1% Triton X100 and 10% DMSO) for 1 h at 4 °C and then placed into a horizontal electrophoresis apparatus filled with freshly made buffer (1 mM Na_2_EDTA. 300 mM NaOH, pH 13.0). After 20 min of preincubation (unwinding of DNA), the electrophoresis was run for 20 min at a fixed voltage of 25 V and 300 mA, which was adjusted by raising or lowering the level of the electrophoresis buffer in the tank. At the end of the electrophoresis, the slides were washed 3 times with the neutralization buffer (Tris-HCl 0.4 M, pH 7.5), stained with 50 mL ethidium bromide (20 mg/mL), and kept in a moisture chamber in the dark at 4 °C until analysis. All the above reported steps were carried out under red light to prevent any additional DNA damage. 

### 4.7. Comet Detection 

The cells were analyzed 24 h after staining at 400× magnification using a fluorescence microscope (Zeiss, Oberkochen, Germany) equipped with a 50-W mercury lamp. The microscopic images revealed circular shapes (undamaged DNA) and “comet-like” shapes in which the DNA had migrated from the head to form a tail (damaged DNA). The extension of each comet was analyzed by a computerized image analysis system (Comet assay II, Perceptive Instruments, Suffolk, UK) that, among several other parameters, gave the “tail moment” that is considered to be the parameter most directly related to DNA damage. In fact, the tail moment is defined as the product of DNA in the tail and the mean distance of its migration in the tail. Calculation of the extent of DNA damage, which was not homogeneous, was based on the analysis of 100 randomly selected comets from each slide, divided into 5 classes according to the tail moment (t.m.) values as follows: class 0 (t.m. < 1; no damage), class 1 (t.m. 1–5; slightly damaged), class 2 (t.m. 5–10; medium damage), class 3 (t.m. 10–20; highly damaged) class 4 (t.m. > 20; completely damaged). The overall score expressed in arbitrary units (A.U.) for each slide ranged from 0 (100% of the comets in class 0) to 400 (100% of the comets in class 4) [65].

### 4.8. Antioxidant Plasma Power Quantification 

Ferric Reducing Ability of Plasma (FRAP) test was used to quantify the antioxidant ability of plasma in mice. This method is based on the measure of the reducing power of plasma. Antioxidants compounds can donate electrons, reducing ferric ion (Fe^3+^) to ferrous ion (Fe^2+^) at low pH. The 2,4,6-tripyridyl-*s*-triazine (TPTZ) in the reaction medium can bind the ferrous ion at low pH. An intense blue colour is developed with an absorption maximum at 593 nm:

Fe(TPTZ)^3+^→Fe(TPTZ)^2+^ (1)

The colour intensity is a measurement of the ferric reducing ability of plasma and, therefore, of its antioxidant capacity. The reagent concentrations were as follows: acetate buffer (300 mM, pH 3.6), TPTZ (10 mM in HCl 40 mM), FeCl_3_·6H_2_O (20 mM) [66]. FRAP reagent was prepared by mixing: 10 volumes of acetate buffer, 1 volume of TPTZ, and 1 volume of FeCl_3_·6H_2_O. Plasma samples were not diluted. In the reaction, 3 mL of FRAP reagent, 100 µL of sample, and 300 µL of deionized water were mixed in a cuvette. This cuvette was shaken and the absorbance recorded for 8 min at 593 nm. The measurements of absorbance were performed in triplicate. A calibration curve was constructed with aqueous solutions of known Fe_2_^+^ concentrations (0, 100, 300, 500, 750, and 1000 µmol/L FeSO_4_ 7H_2_O). Results are expressed as micromoles per liter of Fe^2+^.

### 4.9. Statistical Analysis

To compare different treatments, each other a one-way ANOVA using the GraphPad Prism statistical software program Version 3.0 for Windows (San Diego, CA, USA) was applied. When a significant effect was detected (*p* < 0.05), the mean values were compared using Tukey’s post hoc comparisons. All data are presented as mean ± SD.

## 5. Conclusions

In conclusion, our results indicate that HT exerts beneficial preventive effects against LPS induced systemic inflammation and oxidative damage. The anti-inflammatory properties of this olive oil phenol are mediated by a regulation of several pathways involved in the expression of pro-inflammatory genes and cytokines production, while the anti-oxidant activities are demonstrated by its ability to both prevent oxidative DNA damage and improve total antioxidant power of plasma. Further studies would be need to evaluate if the antioxidant and anti-inflammatory properties of HT are related with the regulation of Nrf2 and NF-kB important transcription factors for genes implicated in oxidative stress and inflammation [67] as observed in other studies [46,68].

Finally, our data encourage further clinical trials to test the use of this molecule, alone and in combination with either the current anti-inflammatory drugs or other polyphenols, in humans for the treatment and/or prevention of various chronic degenerative diseases where inflammation and oxidative stress play an important role. Once the data of the clinical interventions have shown positive results, the HT can be used in the prevention and treatment of important diseases such as rheumatoid arthritis, gastritis, hepatitis, peritonitis and inflammatory bowel diseases.

## Abbreviations

COX2	cyclooxygenase-2
HT	hydroxytyrosol
EVOO	extra-virgin olive oil
FRAP	ferric reducing anti-oxidant power
i.p.	intraperitoneal injection
NSAIDs	non steroideal anti-inflammatory drugs
PBMC	peripheral blood mononuclear cells
qPCR	quantitative PCR
ROS	reactive oxygen species
TNF-α	tumor necrosis factor alfa
